# Failure To Detect Functional Neutrophil B Helper Cells in the Human Spleen

**DOI:** 10.1371/journal.pone.0088377

**Published:** 2014-02-11

**Authors:** Sietse Quirijn Nagelkerke, Daan Jacob aan de Kerk, Machiel Hugo Jansen, Timo Kars van den Berg, Taco Willem Kuijpers

**Affiliations:** 1 Department of Blood Cell Research, Sanquin Research and Landsteiner Laboratory, Amsterdam, The Netherlands; 2 Department of Pediatric Hematology, Immunology and Infectious Diseases, Emma Children's Hospital, Academic Medical Center, Amsterdam, The Netherlands; 3 Department of Experimental Immunology, Academic Medical Center, Amsterdam, The Netherlands; University of Bern, Switzerland

## Abstract

A novel role for human neutrophilic granulocytes was recently described, showing that these cells, upon entering the spleen, can be reprogrammed into a distinct B cell-helper neutrophil phenotype that is capable of eliciting B cell responses such as immunoglobulin secretion, class switch recombination and somatic hypermutation. Using similar protocols, we detected a homogeneous population of CD15^high^CD16^high^ neutrophils in fresh human spleen samples, which did not differ in phenotype and function from blood neutrophils. No phenotypic characteristics of costimulatory nature were detected on splenic or circulating neutrophils, nor could we reproduce the immunoglobulin production of splenic B cells in the presence of splenic neutrophils, although B cell function and neutrophil activity were normal. Independent confirmation of a role for N_BH_ cells is required.

## Introduction

The marginal zone (MZ) in the spleen has a well defined structure and function [1]. It contains a specialized subset of B cells, the marginal zone B (MZ B) cells. A large proportion of the MZ B cells express B-cell receptors that recognize thymus-independent antigens (TI-antigens) [2]. MZ B cells reactive to TI-antigens are able to undergo somatic hypermutation (SHM) [2]–[4] and class switch recombination (CSR) [2], but the co-stimulatory triggers that drive these events are not as clear as for TD-antigens. TLRs on the B cells themselves are known to be involved [5], [6] and mice data show a role for dendritic cells [7] and monocytes [8], but not much is known about the human MZ B cells, which differ from rodents in many aspects [1], [2], [9]. Recently, Puga *et al* described a novel specialized subset of neutrophils in the human spleen capable of stimulating B-cell responses against TI-antigens [10]. These splenic neutrophils or ‘B cell-helper neutrophils’ (N_BH_ cells) were shown to induce IgM production, CSR and SHM in MZ B cells. This capacity was indicated to be specific for splenic neutrophils, as circulating or ‘conventional’ neutrophils (N_C_ cells) were not able to induce such reactions. N_BH_ cells were reported to express B-cell-stimulating molecules, such as CD40L, BAFF, APRIL and IL-21, to induce MZ B cell responses. These neutrophils were divided into 2 distinct subsets: N_BH1_ (CD15^int^CD16^int^) and N_BH2_ (CD15^low^CD16^low^) cells. N_BH2_ cells were most effective in eliciting MZ B cell responses. Since our laboratory has a longstanding interest in neutrophils, combined with the availability of fresh human spleen samples, we tried to characterize these neutrophil subsets further. Our findings indicated that the phenotype of human splenic neutrophils is not different from circulating neutrophils, and their role in MZ B cell activation is limited, if present at all.

## Materials and Methods

### Human Subjects

Spleens were from organ transplant donors (Table S1 in File S1) without clinical signs of infection or inflammation. Written informed consent for organ donation was obtained according to national regulations regarding organ donation. Splenic tissue of the organ donor was obtained during transplantation surgery, as part of the standard diagnostic procedure for HLA-typing, and was transported in University of Wisconsin Fluid at 4°C. In case there was an excess of splenic tissue for diagnostic procedures, this excess of splenic tissue was used in an anonymous fashion for research in the present study, in accordance with the Dutch law regarding the use of rest material for research purposes. Blood samples were rest material from blood samples of organ donors drawn at the time of surgery as a standard diagnostic procedure, or from age matched healthy volunteers. Written informed consent was obtained from all age matched healthy volunteers. The study was approved by the Medical Ethics Committee of the Academic Medical Center and Sanquin in Amsterdam, and was performed in accordance with the Declaration of Helsinki.

### Preparation of cells

Splenocytes were isolated by injecting a piece of spleen at several sites with collagenase buffer (Table S2 in File S1). Connective tissue was removed and the tissue was subsequently incubated in the collagenase buffer for 30 minutes at 37°C. Tissue was then filtered using a 100 µm filter. Subsequently, erythrocytes were lysed with an isotonic ammoniumchloride buffer for 5 minutes at 4°C, after which lysis buffer was washed away. Blood leukocytes were isolated essentially the same way. In a selected set of experiments, spleen tissue was injected with PBS instead of collagenase buffer, and was immediately filtered afterwards.

The NIH3T3 mouse fibroblasts expressing human CD40L have been described previously [11].

### Isolation of neutrophils

Neutrophils were isolated directly from splenocytes or blood leukocytes with EasySep-Human Neutrophil Enrichment Kit (StemCell Technologies), according to the manufacturer's protocol. Isolation was performed at 4°C.

In a selected set of experiments, neutrophils were separated from splenocytes with a Histopaque-1077 gradient (Sigma), followed by purification with the Human Neutrophil Enrichment kit.

### Flowcytometry

Sorting of neutrophils and different B cell subsets was performed on a FACS Aria II machine (BD). Flowcytometric analysis was performed on a FACS Canto II machine (BD). For a list of antibodies see Table S3 in File S1.

### B cell cultures & immunoglobulin determination

Essentially as described in [12], [13]. In brief, MZ B cells were cultured for 7 days at a 1∶1 ratio with stimulating cells, or with 1 µg/ml CpG and 50 U/ml IL-2. Supernatants were tested for secreted IgM and IgG by ELISA using polyclonal rabbit-anti-human IgG and IgM and a serum protein calibrator (Dako) [12], [13].

### Measurement of reactive oxygen species production

NADPH-oxidase activity of neutrophils was measured by hydrogen peroxide (H_2_O_2_) production for 30 minutes in an Amplex Red assay (Invitrogen, Carlsbad, CA, USA), essentially as described before [14].

## Results

**Figure 1:**
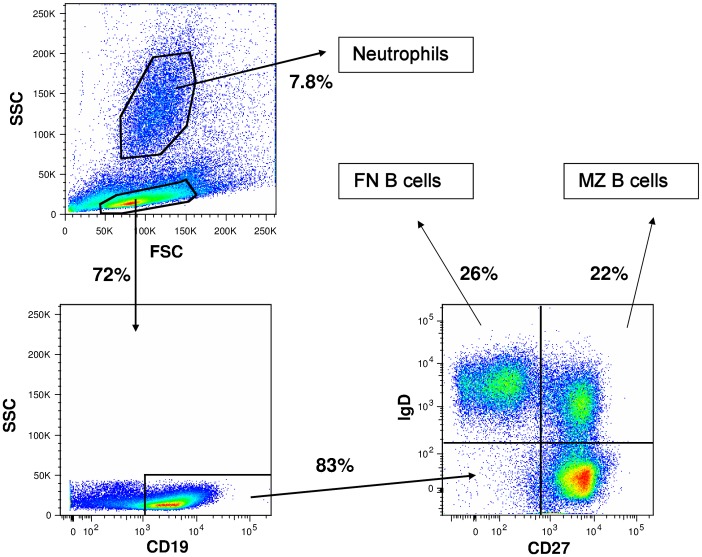
Sorting strategy for sorting Neutrophils and Naïve and Marginal Zone B Cells from splenocytes. Cells were sorted directly from splenocytes stained for CD19, IgD and CD27. Percentages indicate percentage of that population compared to the parent population. FN B Cells: follicular naïve B cells, MZ B cells: marginal zone B cells.

**Figure 2:**
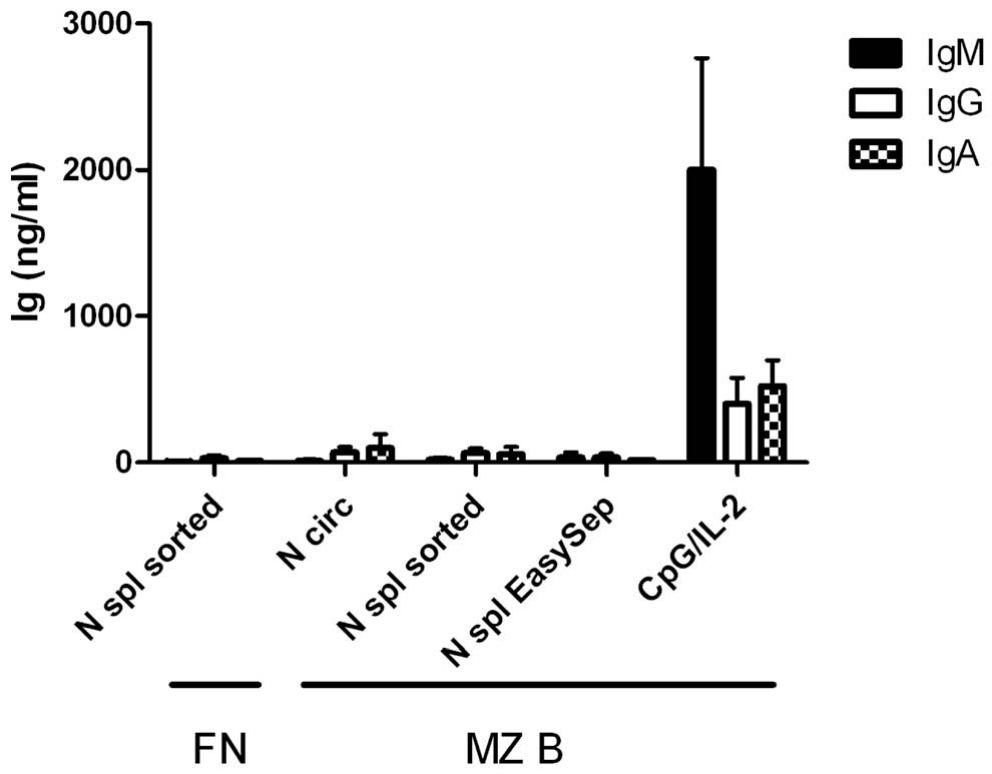
Splenic B cells do not produce immunoglobulin in response to splenic neutrophils. ELISA of IgM, IgG and IgA from splenic marginal zone B cells (MZ B) or follicular naïve B cells (FN), sorted as shown in Figure 1, after co-culture for 7 days with circulating neutrophils (N circ), FACS-sorted spleen neutrophils (N spl sorted), EasySep-isolated spleen neutrophils (N spl EasySep) or CpG/IL-2. Data summarize three independent experiments (error bars, SEM.)

Using fresh spleen sample from healthy organ donors, we isolated MZ B cells (CD19^+^IgD^+^CD27^+^) and Follicular Naïve (FN) B cells (CD19^+^IgD^+^CD27^−^) by FACS-sorting (Figure 1). We isolated splenic neutrophils in two ways: by the EasySep-Neutrophil-Enrichment-Kit as reported by Puga *et al.* [10], or by FACS-sorting from splenocytes as based on their FSC/SSC profile (Figure 1). When co-culturing these B cells with neutrophils, neither of the two splenic neutrophil isolates induced any IgM, IgG or IgA production after 7 days (Figure 2), nor did these splenic neutrophils induce differentiation of B cells to plasmablasts (Figure S1 in File S1) [12]. The MZ B cells were viable and able to produce significant amounts of IgM, IgG and IgA upon stimulation with CpG (Figure 2), concomitant with plasmablast formation (Figure S1 in File S1). Similarly, viability and function of neutrophils was normal, as indicated by a normal production of reactive oxygen species in response to various stimuli (Figure S2 in File S1).

**Figure 3:**
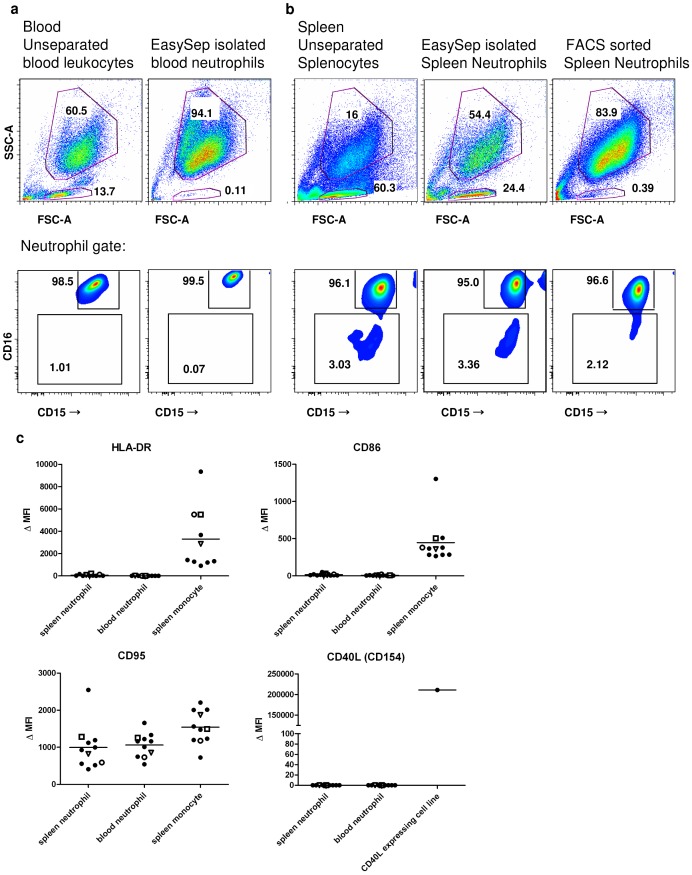
Expression pattern of splenic neutrophils does not differ from the expression pattern of circulating neutrophils. a,b: Upper panels: FSC/SSC pattern of blood (a) or spleen (b) for unseparated leukocyte/splenocyte suspensions, EasySep-isolated neutrophils, and FACS-sorted spleen neutrophils. Numbers indicate percentage of the total population. Lower panels: CD15/CD16 double staining of neutrophils as determined by canonical FSC/SSC. CD15: clone 28, FITC. CD16: clone 3G8, PE. Numbers indicate percentage of the neutrophil population. Data shown are of one spleen donor and one blood donor, and are representative of eight (unseparated), four (EasySep) or three (FACS sorted) independent experiments. **c**. Expression levels of HLA-DR, CD40L, CD86 and CD95 in neutrophils from unseparated leukocyte/splenocyte suspensions, as gated in a and b. Monocytes or a CD40L expressing cell line serve as positive control, gating strategy as in Figure S5a. Data summarize 11 independent experiments. In three of these experiments, the blood and spleen tissue were of the same donor, as indicated by the open symbols (n = 3). The MoAbs used are all commercially available, quality-tested antibodies (Table S3), with the same CD15 and CD16 clones as used by Puga *et al* [10]. Δ MFI: median fluorescence intensity minus median fluorescence intensity of appropiate isotype control.

Whereas the purity of FACS-sorted splenic neutrophils and EasySep-isolated circulating neutrophils was excellent (<0.5% lymphocytes), the EasySep-isolated splenic neutrophils consistently contained a large population (ranging from 10 to 25%) of lymphoid cells (Figure 3a and Figure S3a in File S1). These contaminating cells were mainly CD20^+^/CD27^+^ B cells, containing both IgM^pos^ and IgG^pos^ cells (Figure S3b in File S1). A pre-enrichment step by density gradient centrifugation did not remove this population of B cells (Figure S4 in File S1).

Upon further analysis of these freshly isolated splenocytes, we tested EasySep-isolated and FACS-sorted neutrophils as well as unseparated splenocytes for expression of various surface antigens. In contrast to the findings of Puga *et al*, the majority (>96%) of these cells showed high expression levels of CD15 and CD16, both in spleen and blood (Figure 3a,b and Figure S5e in File S1). We did not detect any surface expression of CD40L, CD86, HLA-DR or increased levels of CD95 on these splenic neutrophils (Figure 3c and Figure S5a–d in File S1).

## Discussion

We could not reproduce the stimulation of immunoglobulin production in human MZ B cells by splenic neutrophils, despite the fact that both the MZ B cells and the neutrophils we isolated were fully viable and functional. Possible explanations for the discrepancy with the findings of Puga *et al.* [10] include differences in the protocols for obtaining spleen tissue and isolating spleen cells, discussed further below. Of particular concern is our finding of a consistent contamination with B cells in EasySep-isolated splenic neutrophils. The notion that N_BH_ cells can induce CSR in MZ B cells in *in vitro* cultures of 1 week is solely based on cultures with EasySep-isolated splenic neutrophils [10]. Our data show that splenic neutrophil samples isolated by the Easysep-Neutrophil-Enrichment-Kit, as opposed to blood samples, consistently contained a contaminating B-cell population that was partly IgG^pos^. Such a population will contain germline I_γ_1-C_γ_1 or I_γ_2-C_γ_2 transcripts, and I_γ_-C_μ_ switch circles, in itself. Presence of these transcripts in cultures of MZ B cells stimulated with B-cell contaminated neutrophils therefore does not prove the induction of CSR. The Easysep-Neutrophil-Enrichment-Kit can be used for isolation of blood neutrophils, but the isolation of neutrophils from other sources is not mentioned by the manufacturer. Purity of EasySep-isolated splenic neutrophil fractions was not shown by Puga *et al*.

Furthermore, we could not find the characteristic phenotypes of N_BH1_ and N_BH2_ cells as described [10]. Slight differences in the protocol for obtaining splenocytes may form an obvious explanation. Regarding the isolation protocol, the only difference between the protocols consisted of an incubation step with collagenase and DNAse. However, when we isolated splenocytes by perfusion with PBS only [10], expression of HLA-DR, CD40L, CD86 or CD95 on splenic neutrophils was still absent (Figure S6 in File S1), showing that the treatment with collagenase did not influence expression of these molecules. Treatment of splenic tissue with collagenase and DNAse is a widely used method [15] that is used to obtain also cells that are embedded in or have infiltrated tissue matrix, as N_BH_ cells supposedly do, which is the reason we have chosen to include it in our protocol.

Instead, differences in the protocol for obtaining splenic tissue may have been critical. We used only fresh spleen samples that were obtained from heart-beating organ donors and were transported at 4°C in a specialized medium for organ conservation. Neutrophils are known to be easily activated during transport and isolation, resulting in the cleavage of CD16 from the cell surface [16]–[18]. Such events may have led to the reported phenotypes of N_BH_ cells. In fact, the finding of Puga *et al.* that splenic samples contained only N_BH_ cells in the absence of N_C_ cells is surprising in itself, as the spleen is highly vascularized and must contain lots of circulating blood cells. Further evidence for the fact that handling of splenic tissue prior to isolation can influence the phenotype of neutrophils comes from the intriguing finding of Puga *et al.* that inflamed splenic tissue only contained the (less active) N_BH1_ cells, as these were isolated from frozen or paraffin-embedded tissue [10]. In our case, we regard it unlikely that unfavorable circumstances during isolation of our neutrophils have prevented us from finding the phenotype of specialized N_BH1_ and N_BH2_ cells in 11 spleens, which consistently revealed neutrophil populations very similar to circulating neutrophils.

Apart from technical issues, the findings of specific splenic neutrophil functions led to a lot of confusion and debate, because patients with neutropenia or chronic granulomatous disease are not known to present with clinical infections associated with B cell deficiencies [19], [20]. Concluding, we did not find evidence for the reprogramming of circulating neutrophils into distinct N_BH1_ and N_BH2_ cells in the human spleen *in vivo*, nor did we find evidence for the major role of neutrophils in MZ B cell stimulation that was proposed by Puga *et al*. We encourage others to attempt to reproduce these experiments in order to establish whether splenic N_BH_ cells exist and play a role in B-cell activation. In our opinion, further research defining the triggers that drive B cell responses against TI-antigens in the human situation should not be solely focused on neutrophils, but should also consider other potentially involved cell populations such as monocytes or dendritic cells.

## Supporting Information

File S1 **Includes Figures S1–S4 and Tables S1–S3.** Figure S1. Splenic marginal zone B cells are able to differentiate into plasmablasts in response to CpG/IL2, but not in response to blood or spleen neutrophils. FACS plot of CD27/CD38 double staining of CD20^pos^ B cells cultured for 7 days with indicated stimuli. Numbers indicate percentage of the total B cell population. Figure S2. Measurement of neutrophil reactive oxygen species in response to different stimuli. Production of reactive oxygen species by neutrophils from spleen and blood. RFU: relative fluorescence units, which are a derivative of H_2_O_2_ production. Zymosan 1 mg/ml; STZ: serum-treated zymosan 1 mg/ml; PMA: Phorbol 12-Myristate 13-Acetate 100 ng/ml; fMetLeuPhe 1 µM; PAF: platelet-activating factor 1 µM. Spleen n = 2, blood n = 3. Error bars represent standard deviation. Figure S3. Purity analysis of different neutrophil isolates. a. FSC/SSC plot and May-Grünwald/Giemsa stained cytospins of different neutrophil isolates. Neutrophils (upper gate) and lymfocytes (lower gate) are gated according to canonical FSC/SSC pattern. Numbers indicate percentages of total events. Original magnification of cytospins 540x. Data are representative of four independent experiments. b. Characterisation of the lymfocyte population contaminating the EasySep-isolated spleen neutrophils. Numbers indicate percentages of the lymfocyte population. Figure S4. Ficoll density gradient centrifugation prior to EasySep isolation does not remove the contaminating B cell population from EasySep-isolated splenic neutrophils. FSC/SSC pattern of splenic neutrophils separated either directly from splenocytes with the Human Neutrophil Enrichment kit (left), or separated from splenocytes with a Histopaque-1077 gradient followed by purification with the Human Neutrophil Enrichment kit (right). Numbers indicate percentage of total events. Data are representative of 2 independent experiments. Figure S5. Expression patterns of splenic neutrophils do not differ from their circulating counterpart. a.Gating strategy for neutrophils (upper gate) and monocytes (lower gate) by canonical FSC/SSC pattern in blood and spleen. The monocyte gate may include small percentages of other cells, especially in spleen, and serves only to show a positive control for the antibodies used. b.Staining of HLA-DR, CD86, CD95 and CD40L of splenic neutrophils as gated in Figure 2 and splenic monocytes as gated in Figure S5a. Data are representative of 11 independent experiments. Black lines: Staining with monoclonal antibody. Gray shading: isotype control. c. Staining of HLA-DR, CD86, CD95 and CD40L of blood neutrophils as gated in Figure 2 and blood monocytes as gated in Figure S5a. Data are representative of 11 independent experiments. Black lines: Staining with monoclonal antibody. Gray shading: isotype control. d.Staining of CD40L in human CD40L expressing fibroblast cell line. Black lines: Staining with monoclonal antibody. Gray shading: isotype control. e. CD15/CD16 double staining of blood and splenic neutrophils, as gated in Figure 2. Antibodies used: CD15: clone HI98, FITC. CD16: clone 3G8, APC. Data shown are of blood and splenic neutrophils from a single donor, and representative of two other experiments with unpaired samples. Figure S6. Incubation with collagenase buffer does not influence expression profile of neutrophils. Expression levels of HLA-DR, CD40L, CD86 and CD95 in neutrophils and monocytes from unseparated splenocyte suspensions, gated as in Figure S5a. Splenocytes suspensions were obtained either by perfusion with ‘collagenase buffer’ followed by 30 minute incubation at 37°C, or by perfusion with PBS alone with direct further processing. Data summarize 2 independent experiments, open triangles represent 1 donor, closed circles represent the other donor. Δ MFI: median fluorescence intensity minus median fluorescence intensity of appropiate isotype control. Table S1. Origin and characteristics of tissue samples. Table S2. Contents of collagenase buffer. Table S3. Antibodies used in flow cytometry.
(PDF)
