# Supplementary material for: Failure To Detect Functional Neutrophil B Helper Cells in the Human Spleen
Source: PLoS One. 2014 Feb 11;9(2):e88377. doi: 10.1371/journal.pone.0088377 (PMC3921168; doi:10.1371/journal.pone.0088377)
Supplement: File S1 — Includes Figures S1–S4 and Tables S1–S3. Figure S1. Splenic marginal zone B cells are able to differentiate into plasmablasts in response to CpG/IL2, but not in response to blood or spleen neutrophils. FACS plot of CD27/CD38 double staining of CD20pos B cells cultured for 7 days with indicated stimuli. Numbers indicate percentage of the total B cell population. Figure S2. Measurement of neutrophil reactive oxygen species in response to different stimuli. Production of reactive oxygen species by neutrophils from spleen and blood. RFU: relative fluorescence units, which are a derivative of H2O2 production. Zymosan 1 mg/ml; STZ: serum-treated zymosan 1 mg/ml; PMA: Phorbol 12-Myristate 13-Acetate 100 ng/ml; fMetLeuPhe 1 µM; PAF: platelet-activating factor 1 µM. Spleen n = 2, blood n = 3. Error bars represent standard deviation. Figure S3. Purity analysis of different neutrophil isolates. a. FSC/SSC plot and May-Grünwald/Giemsa stained cytospins of different neutrophil isolates. Neutrophils (upper gate) and lymfocytes (lower gate) are gated according to canonical FSC/SSC pattern. Numbers indicate percentages of total events. Original magnification of cytospins 540x. Data are representative of four independent experiments. b. Characterisation of the lymfocyte population contaminating the EasySep-isolated spleen neutrophils. Numbers indicate percentages of the lymfocyte population. Figure S4. Ficoll density gradient centrifugation prior to EasySep isolation does not remove the contaminating B cell population from EasySep-isolated splenic neutrophils. FSC/SSC pattern of splenic neutrophils separated either directly from splenocytes with the Human Neutrophil Enrichment kit (left), or separated from splenocytes with a Histopaque-1077 gradient followed by purification with the Human Neutrophil Enrichment kit (right). Numbers indicate percentage of total events. Data are representative of 2 independent experiments. Figure S5. Expression patterns of splenic neutrophils do not di [file pone.0088377.s001.pdf]

Figure S1

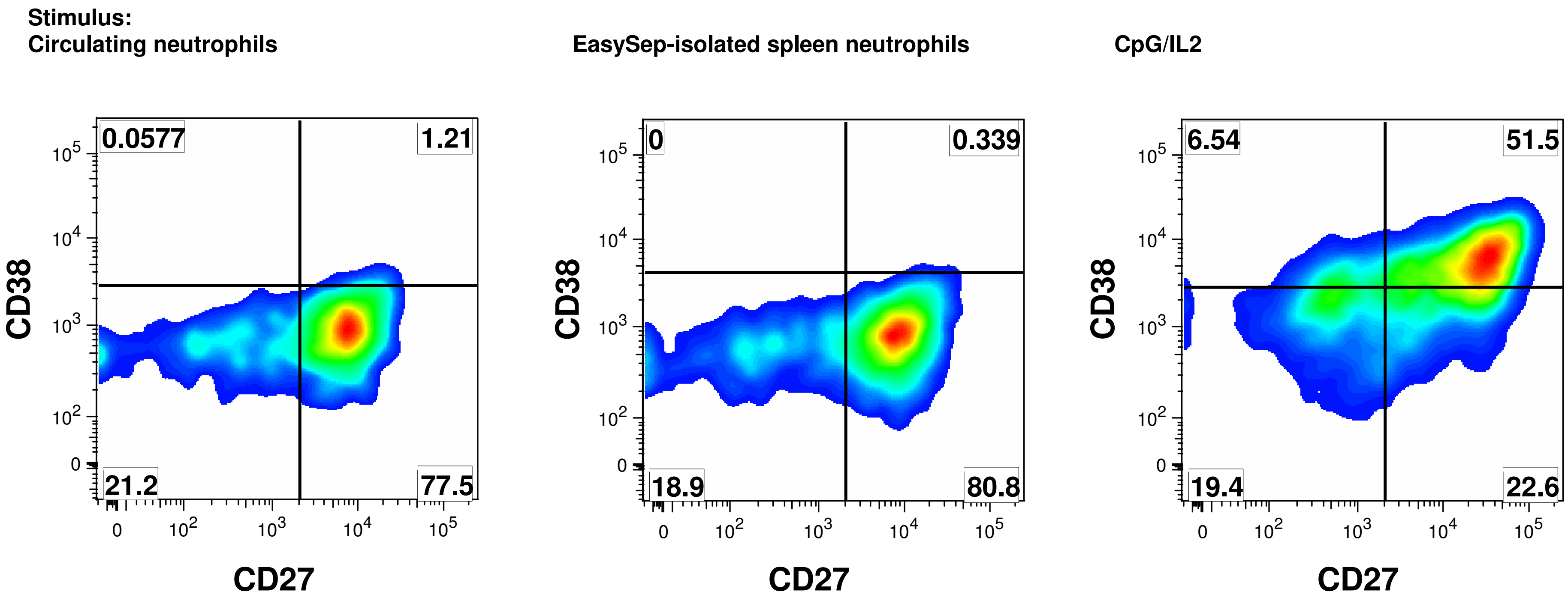

Stimulus:
Circulating neutrophils
EasySep-isolated spleen neutrophils
CpG/IL2
0.0577
1.21
21.2
77.5
0
0.339
18.9
80.8
6.54
51.5
19.4
22.6
CD38
CD27

Figure S2

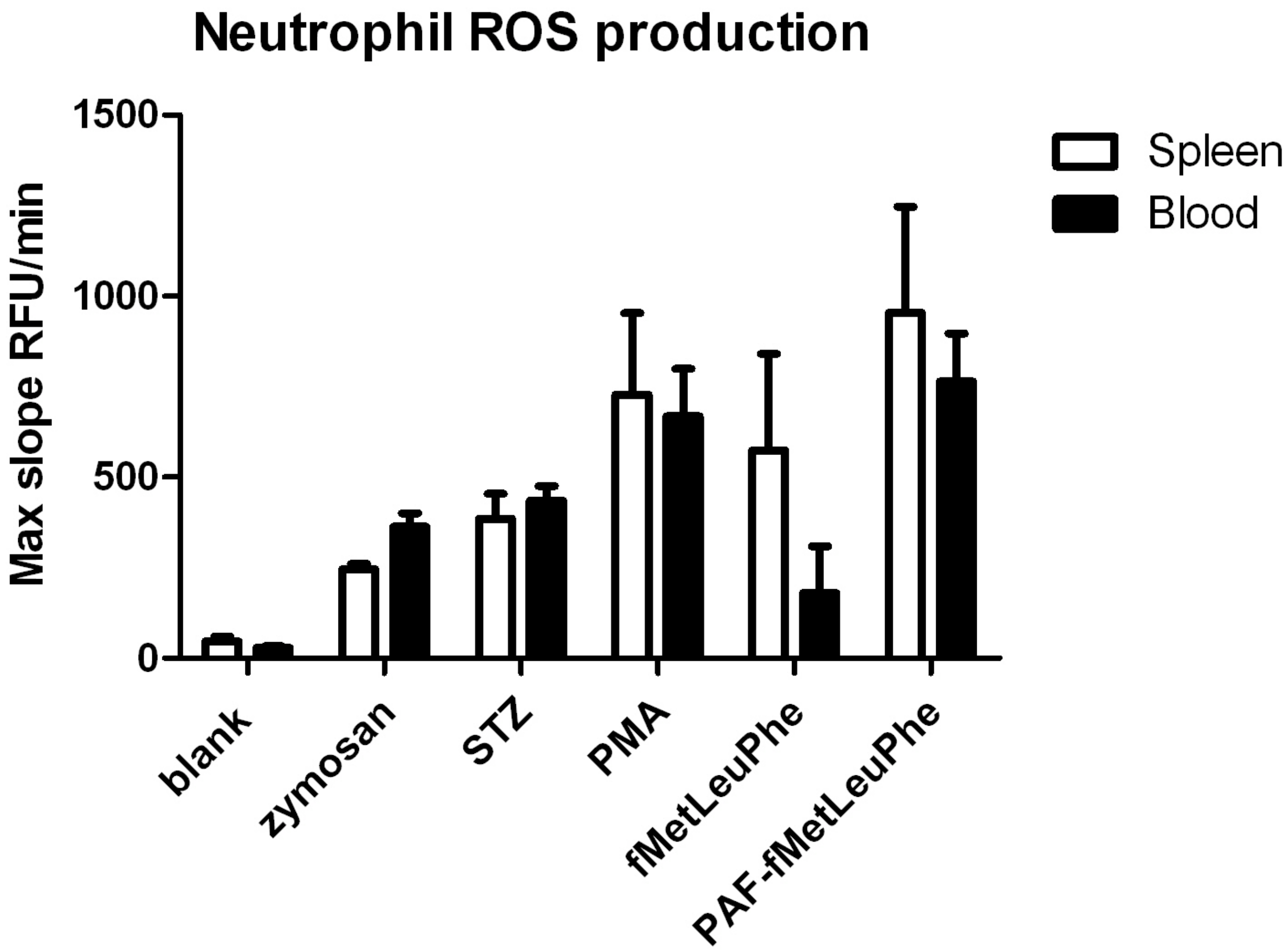
Neutrophil ROS production
Max slope RFU/min
1500
1000
500
0
Spleen
Blood
blank
zymosan
STZ
PMA
fMetLeuPhe
PAF-fMetLeuPhe

Figure S3a

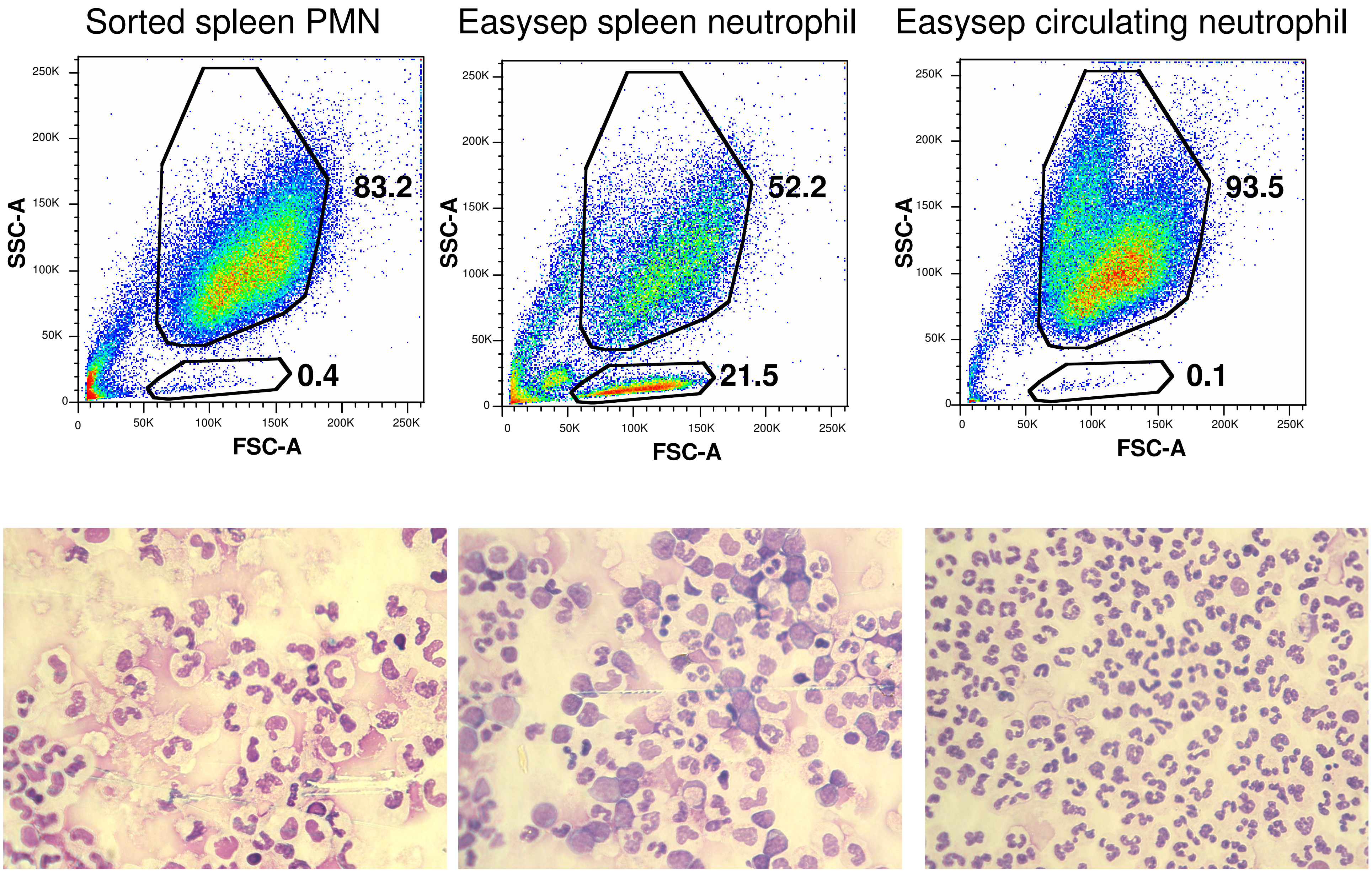

Sorted spleen PMN
Easysep spleen neutrophil
Easysep circulating neutrophil
83.2
0.4
52.2
21.5
93.5
0.1
SSC-A
FSC-A
250K
200K
150K
100K
50K
0

Figure S3b

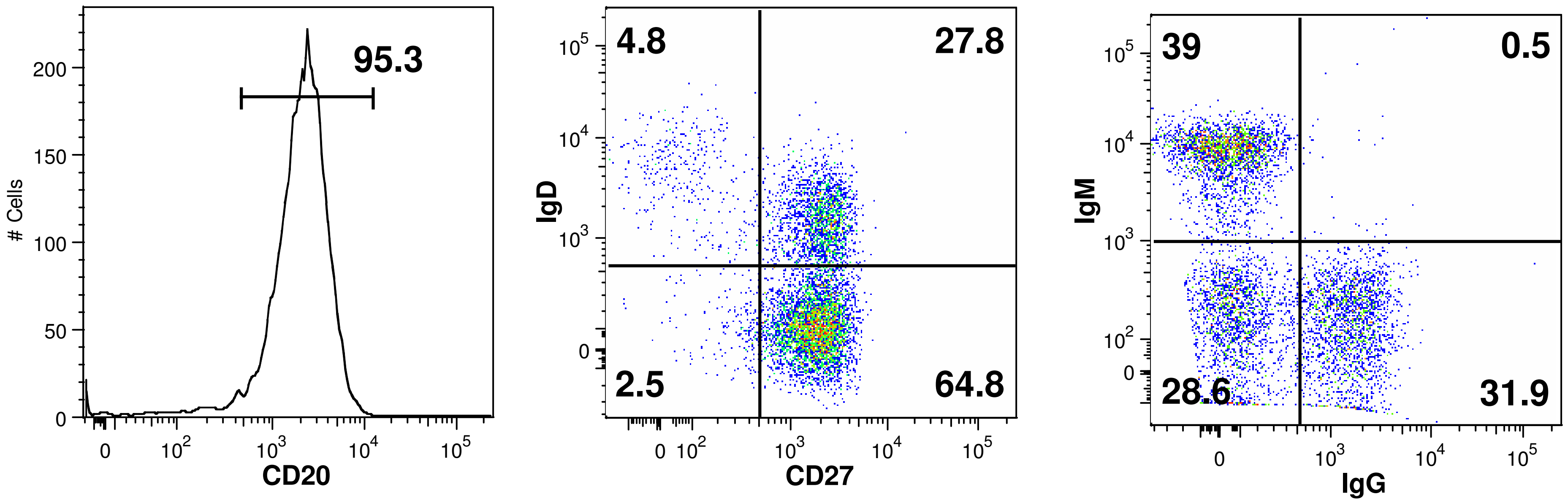

Figure S4

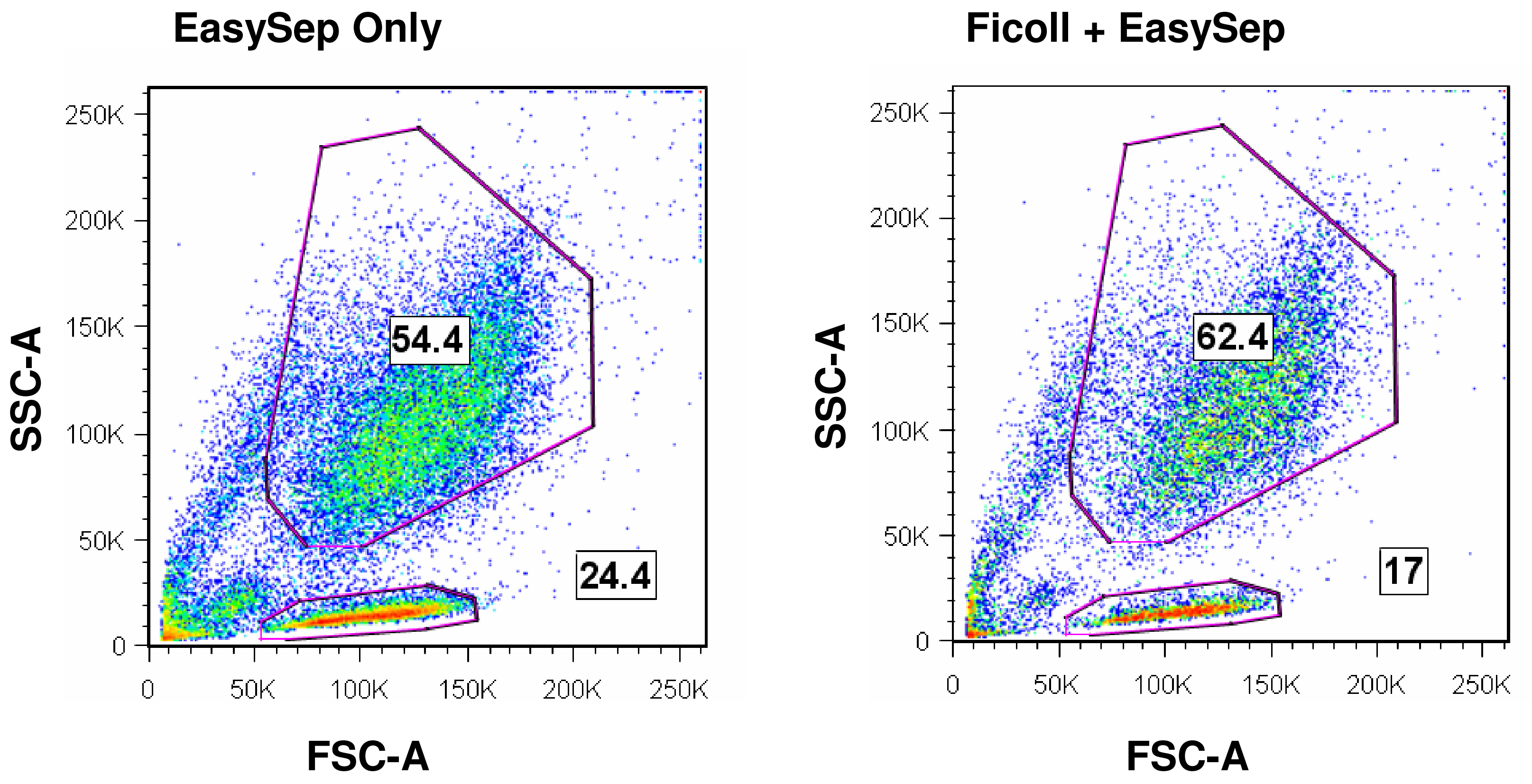
EasySep Only
Ficoll + EasySep
SSC-A
FSC-A
250K
200K
150K
100K
50K
0
50K
100K
150K
200K
250K
54.4
24.4
62.4
17

Figure S5a

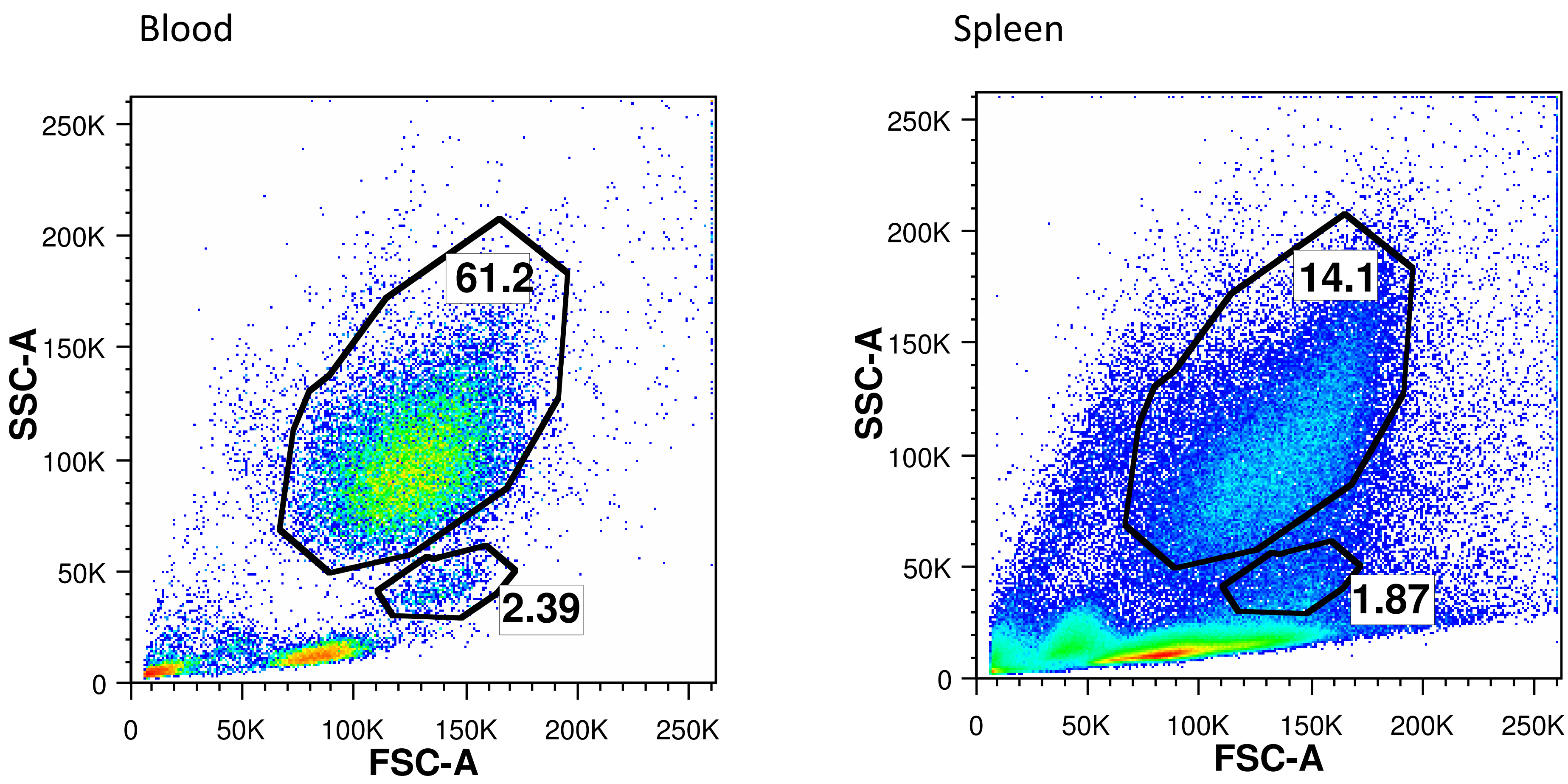
Blood
250K
200K
150K
100K
50K
0
SSC-A
0
50K
100K
150K
200K
250K
FSC-A
61.2
2.39
Spleen
250K
200K
150K
100K
50K
0
SSC-A
0
50K
100K
150K
200K
250K
FSC-A
14.1
1.87

Figure S5b

Spleen Neutrophils

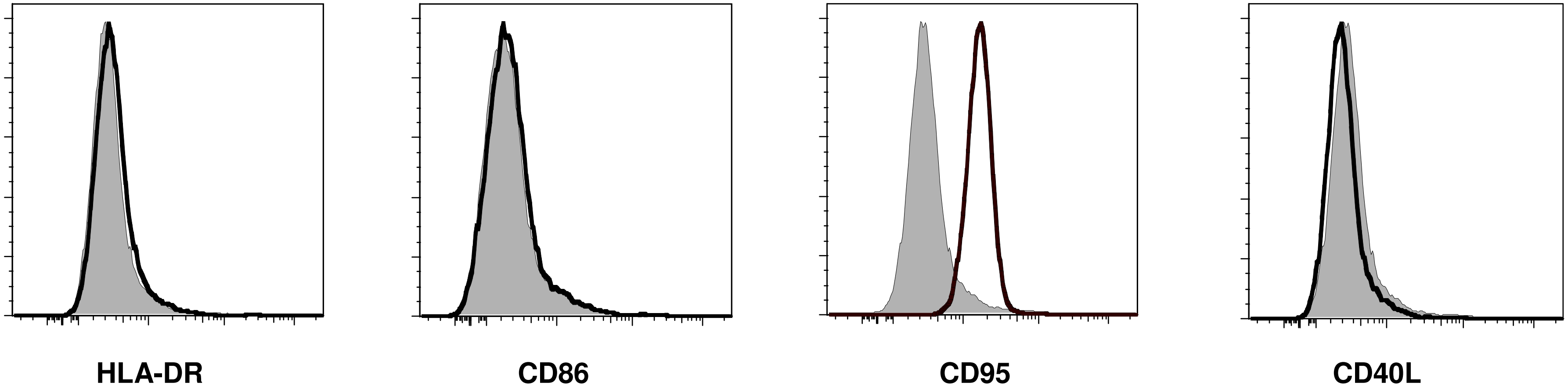

Spleen Monocytes

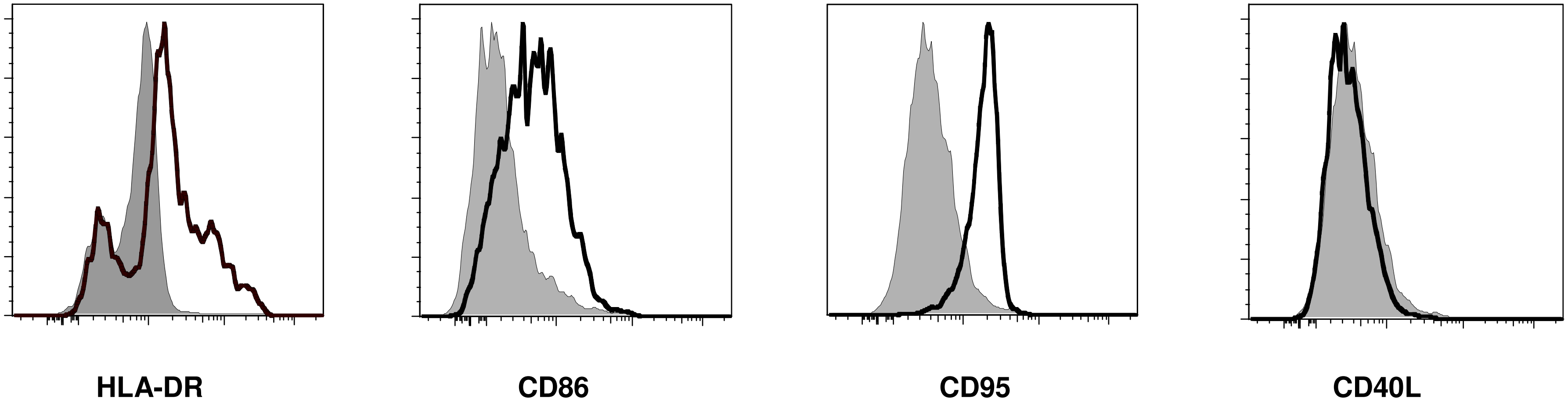

Figure S5c

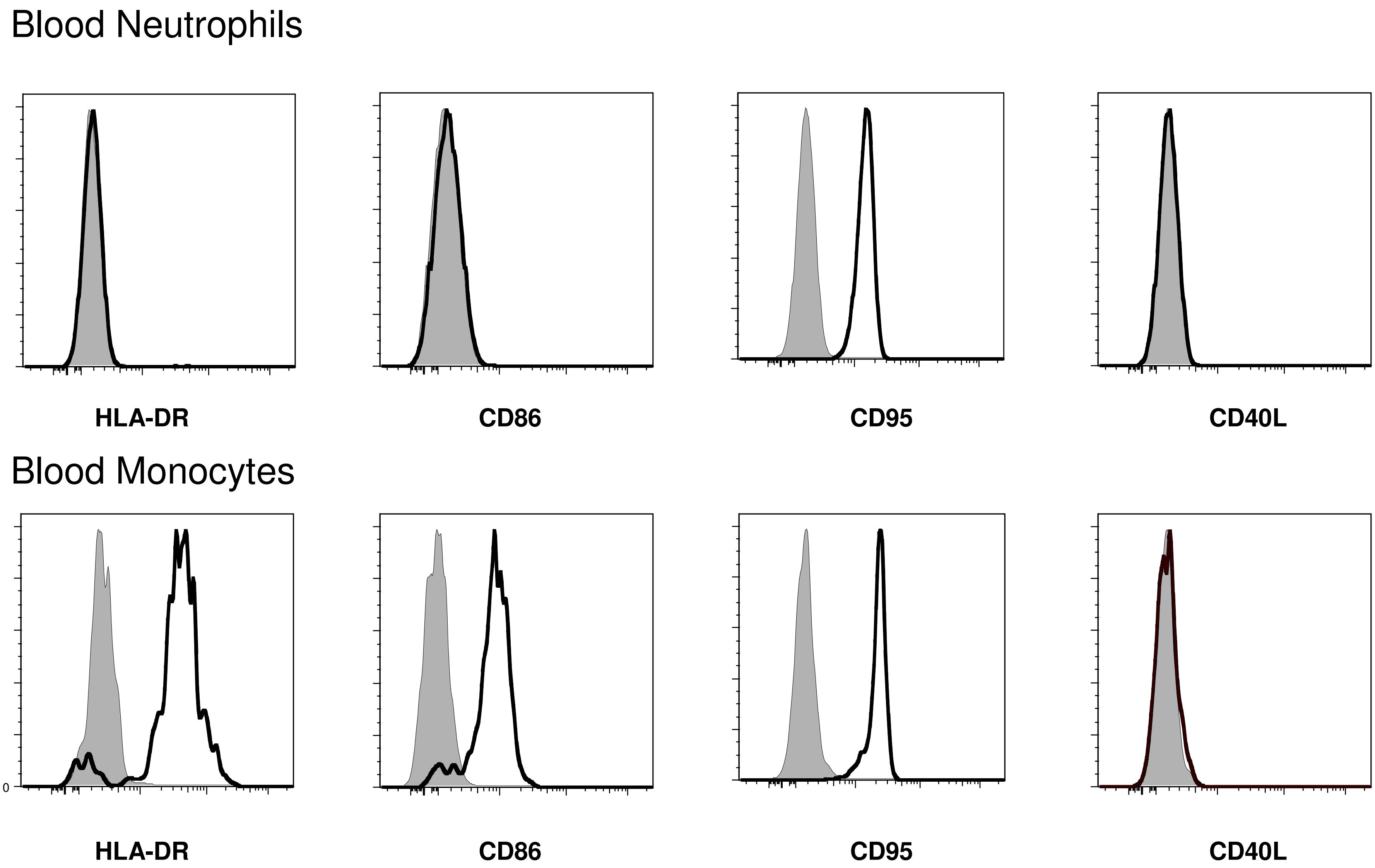

Blood Neutrophils
HLA-DR
CD86
CD95
CD40L
Blood Monocytes
HLA-DR
CD86
CD95
CD40L

Figure S5d

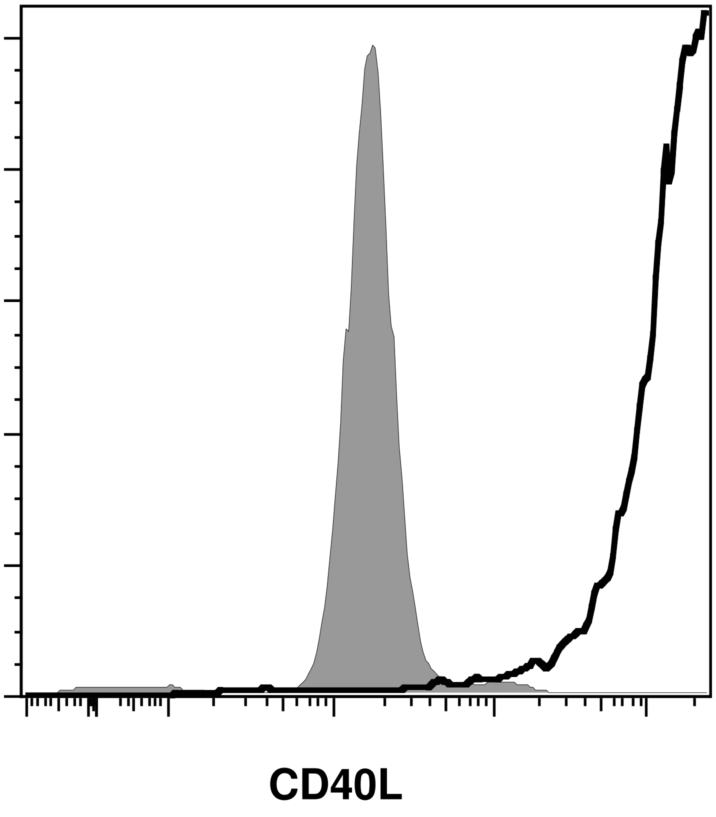

Figure S5e

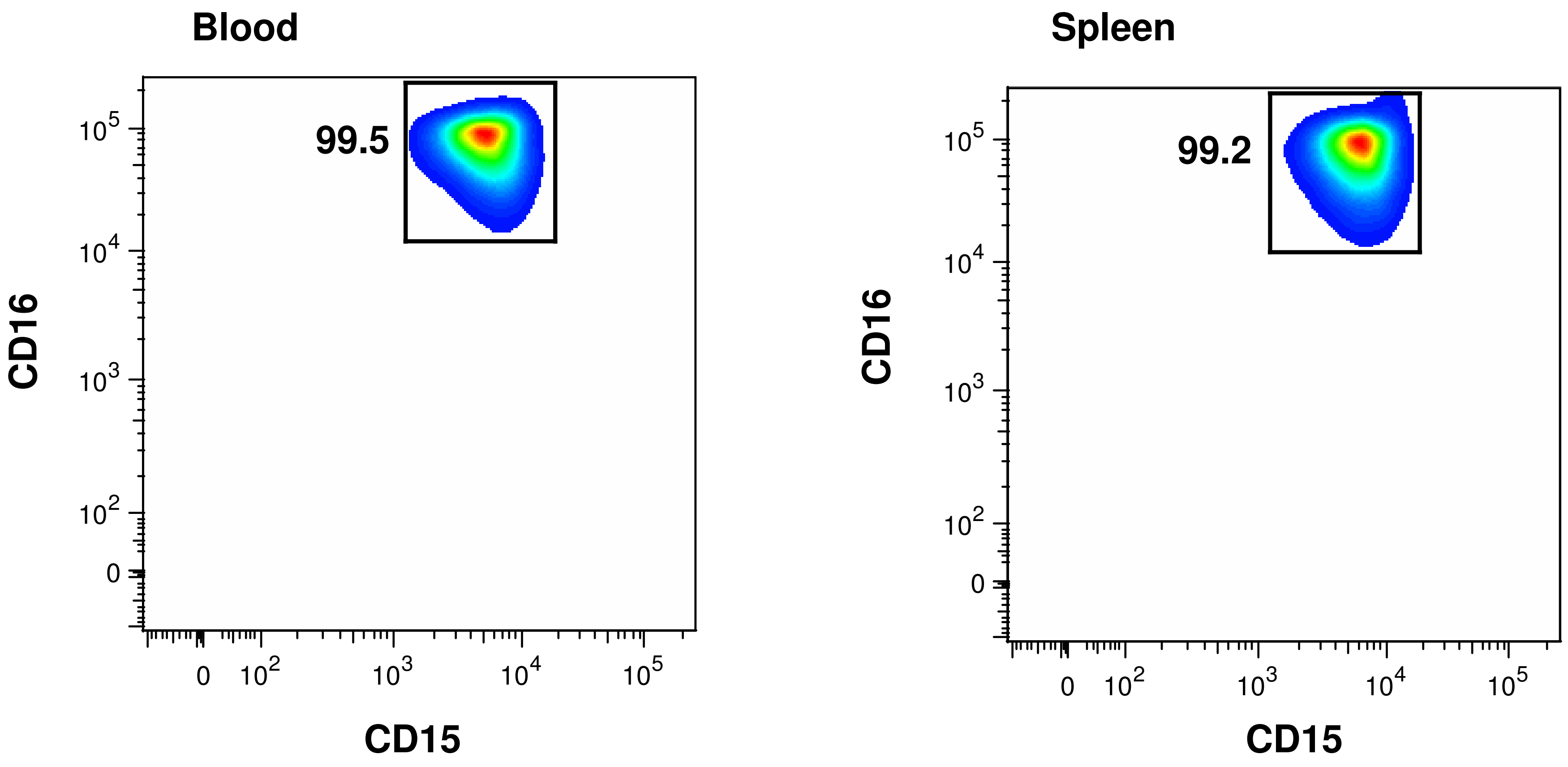

Figure S6

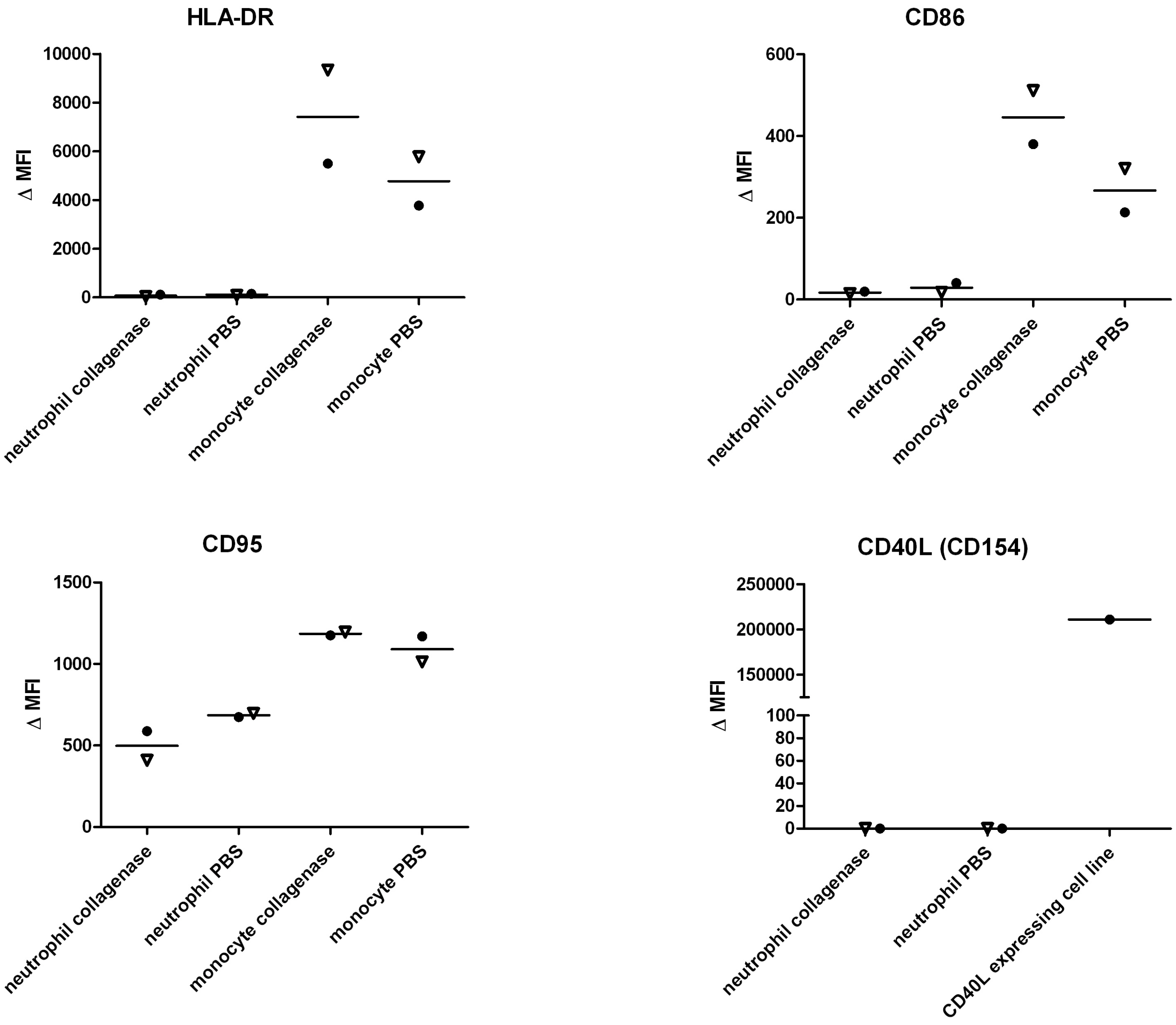

HLA-DR
Δ MFI
10000
8000
6000
4000
2000
0
neutrophil collagenase
neutrophil PBS
monocyte collagenase
monocyte PBS
CD86
Δ MFI
600
400
200
0
neutrophil collagenase
neutrophil PBS
monocyte collagenase
monocyte PBS
CD95
Δ MFI
1500
1000
500
0
neutrophil collagenase
neutrophil PBS
monocyte collagenase
monocyte PBS
CD40L (CD154)
Δ MFI
250000
200000
150000
100
80
60
40
20
0
neutrophil collagenase
neutrophil PBS
CD40L expressing cell line

Table S1

Origin and characteristics of tissue samples

Age donor	Sex donor	Cause of death	spleen tissue obtained
58	male	unknown, approved organ donor	during transplantation surgery
62	male	unknown, approved organ donor	during transplantation surgery
59	male	unknown, approved organ donor	during transplantation surgery
?	female	unknown, approved organ donor	during transplantation surgery
32	male	unknown, approved organ donor	during transplantation surgery
34	male	unknown, approved organ donor	during transplantation surgery
68	male	unknown, approved organ donor	during transplantation surgery
76	male	unknown, approved organ donor	during transplantation surgery
66	male	unknown, approved organ donor	during transplantation surgery
64	male	unknown, approved organ donor	during transplantation surgery
65	male	unknown, approved organ donor	during transplantation surgery

Table S2

Contents of collagenase buffer

Contents	Supplier
100 U/ml collagenase CLSPA	Worthington Biochemical Corporation
2 Kunitz Units/ml DNAse (Deoxyribonuclease I, bovine recombinant,)	Sigma-Aldrich
0.5 µg/ml Aggrastat	MSD
1 mg/ml Glucose	Sigma-Aldrich
1mM Calcium Chloride	Merck

Table S3

Antibodies used in flow cytometry

Antigen	Label	Isotype	Clone	Manufacturer	Use
CD15	FITC	Mouse IgM	28	Southern Biotech	Flow cytometry
	FITC	Mouse IgM	HI98	BD Biosciences	Flow cytometry
CD16	PE	Mouse IgG1	3G8	BD Biosciences	Flow cytometry
	APC	Mouse IgG1	3G8	Bio-connect	Flow cytometry
CD19	Alexa Fluor700	Mouse IgG1	HB19	BD Biosciences	Flow cytometry
CD20	PerCP Cy5.5	Mouse IgG1	L27	BD Biosciences	Flow cytometry
CD27	APC	Mouse IgG1	L128	BD Biosciences	Flow cytometry
CD38	Pe-Cy7	Mouse IgG1	HIT2	BD Biosciences	Flow cytometry
CD86	PerCP Cy5.5	Mouse IgG1	2331 (FUN-1)	BD Biosciences	Flow cytometry
CD95	FITC	Mouse IgG1	FAS19	PeliCluster	Flow cytometry
CD154 (CD40L)	FITC	Mouse IgG1	TRAP1	BD Biosciences	Flow cytometry
HLA-DR	FITC	Mouse IgG2a	G46-6	BD Biosciences	Flow cytometry
IgD	PE	Mouse IgG2a	IA6-2	BD Biosciences	Flow cytometry
IgM	FITC	Rabbit F(ab')2	Polyclonal	DAKO	Flow cytometry
IgG	PE	Mouse IgG1	G18-145	BD Biosciences	Flow cytometry
